# A multimodal approach to automated hierarchical assessment of bulbar involvement in amyotrophic lateral sclerosis

**DOI:** 10.3389/fneur.2024.1396002

**Published:** 2024-05-21

**Authors:** Panying Rong, Lindsey Heidrick, Gary L. Pattee

**Affiliations:** ^1^Department of Speech-Language-Hearing: Sciences and Disorders, University of Kansas, Lawrence, KS, United States; ^2^Department of Hearing and Speech, University of Kansas Medical Center, Kansas City, KS, United States; ^3^Neurology Associate P.C., Lincoln, NE, United States

**Keywords:** bulbar involvement, multimodal assessment, instrumental measurement, computational technique, surface electromyography, speech, neurodegenerative disease, machine learning

## Abstract

**Introduction:**

As a hallmark feature of amyotrophic lateral sclerosis (ALS), bulbar involvement leads to progressive declines of speech and swallowing functions, significantly impacting social, emotional, and physical health, and quality of life. Standard clinical tools for bulbar assessment focus primarily on clinical symptoms and functional outcomes. However, ALS is known to have a long, clinically silent prodromal stage characterized by complex subclinical changes at various levels of the bulbar motor system. These changes accumulate over time and eventually culminate in clinical symptoms and functional declines. Detection of these subclinical changes is critical, both for mechanistic understanding of bulbar neuromuscular pathology and for optimal clinical management of bulbar dysfunction in ALS. To this end, we developed a novel multimodal measurement tool based on two clinically readily available, noninvasive instruments—facial surface electromyography (sEMG) and acoustic techniques—to hierarchically assess seven constructs of bulbar/speech motor control at the neuromuscular and acoustic levels. These constructs, including prosody, pause, functional connectivity, amplitude, rhythm, complexity, and regularity, are both mechanically and clinically relevant to bulbar involvement.

**Methods:**

Using a custom-developed, fully automated data analytic algorithm, a variety of features were extracted from the sEMG and acoustic recordings of a speech task performed by 13 individuals with ALS and 10 neurologically healthy controls. These features were then factorized into 10 composite outcome measures using confirmatory factor analysis. Statistical and machine learning techniques were applied to these composite outcome measures to evaluate their reliability (internal consistency), validity (concurrent and construct), and efficacy for early detection and progress monitoring of bulbar involvement in ALS.

**Results:**

The composite outcome measures were demonstrated to (1) be internally consistent and structurally valid in measuring the targeted constructs; (2) hold concurrent validity with the existing clinical and functional criteria for bulbar assessment; and (3) outperform the outcome measures obtained from each constituent modality in differentiating individuals with ALS from healthy controls. Moreover, the composite outcome measures combined demonstrated high efficacy for detecting subclinical changes in the targeted constructs, both during the prodromal stage and during the transition from prodromal to symptomatic stages.

**Discussion:**

The findings provided compelling initial evidence for the utility of the multimodal measurement tool for improving early detection and progress monitoring of bulbar involvement in ALS, which have important implications in facilitating timely access to and delivery of optimal clinical care of bulbar dysfunction.

## 1 Introduction

As one of the most devastating and fatal neurodegenerative diseases, amyotrophic lateral sclerosis (ALS) results from the degeneration of upper and lower motor neurons (UMN/LMN) along with other cells in both the central and peripheral nervous systems. ALS impacts normal functioning of the neuromuscular system, leading to weakness, atrophy, fasciculation, and loss of active control over skeletal muscles (1–4). These changes usually start focally in a specific body region and progress to other regions over time. As a disease of uncertain etiology, ALS is characterized by substantial heterogeneity. Clinically, about 25%–30% of patients are diagnosed with a bulbar onset, with initial symptoms manifesting as speech and/or swallowing disorders; the rest of patients are diagnosed with a spinal onset, with initial symptoms most commonly starting in the limb region (5). Despite the variability in onset site, most patients develop bulbar symptoms as their disease progresses (6). Bulbar dysfunction has a devastating impact on social, emotional, and physical health, significantly reducing the quality of life (7).

As with all neurodegenerative diseases, ALS is characterized by a long, clinically silent prodromal phase that precedes the clinical symptom onset (8). During this phase, the motor systems have already experienced notable subclinical changes, which after years to decades of accumulation eventually culminate in clinical symptoms and functional declines. For the bulbar motor system, a mounting body of evidence has revealed subclinical changes at various levels (e.g., neuromuscular, kinematic), which occur long before the presentation of dysarthria and dysphagia symptoms (9–11). Accurate detection of these subclinical changes has important scientific and clinical implications. Scientifically, these changes provide a window into the underlying bulbar pathophysiology and thus hold mechanistic relevance. Clinically, the presentation of bulbar symptoms is known to associate with faster progression, poor prognosis, and shorter survival (12–15). Thus, early detection of subclinical bulbar changes before the clinical symptom onset can afford clinicians more time to engage patients (and their caregivers) into the conversation about the impending bulbar functional declines and the available clinical management options [e.g., voice banking, augmentative and alternative communication (AAC)]. Such conversation is essential for informed decision making on developing an optimal care plan to slow functional declines, preserve quality of life, and maximize patient benefits. In addition to early detection, monitoring of subclinical changes related to the progression of bulbar involvement also has important clinical implications, which would guide clinicians in selecting and basing intervention on the evolving needs of the patient over their disease course.

Despite the scientific and clinical significance, standard clinical tools in the current bulbar assessment practice, including neurological exams, patient- and clinician-based symptom reports, and functional speech and swallowing assessments, focus primarily on clinical symptoms and functional outcomes; these tools in general lack sensitivity and reliability for detecting subclinical changes in the bulbar motor system, especially during the prodromal stage (2, 7, 16). Such limitations posit a major challenge to early detection, monitoring, and optimal management of bulbar involvement in ALS. To address these limitations, a growing body of research has been directed toward developing objective bulbar measurement tools. These tools build upon different instruments ranging from lab-based equipment, such as electromagnetic or optical motion tracking systems for recording orofacial kinematics, to personal digital devices, such as smartphones and related apps for audiovisual recording. The wide range of instruments allows various modalities of data to be collected, based on which a variety of objective measures have been developed to assess and characterize subclinical changes in the bulbar motor system.

From the acoustic modality, spectral and cepstral features are commonly used to assess vowel distortion, consonant imprecision, abnormal voice quality (e.g., dysphonia), and prosodic deficits during speech (17–30). In addition, time-domain features (e.g., rate and variability of vocalic, consonantal, and syllabic intervals, pause duration) have been employed to assess rhythmic disturbances and abnormal pause patterns during speech (31–33). From the kinematic modality, both pointwise and trajectory-based measures have been derived to characterize the positioning, movement, and coordination of orofacial structures (e.g., tongue, jaw, lips). These measures, including range of motion, displacement, speed/velocity, acceleration, jerk, cumulative path, area, asymmetry, variability, and spatiotemporal coupling, provide targeted assessment of reduced, slowed, jerky, asymmetrical, irregular, and dyscoordinated orofacial motion, as well documented in the motor speech disorders literature (11, 24, 25, 30, 34–42). Such speech-based acoustic and kinematic measures have shown promise for detecting clinically indiscernible subclinical bulbar involvement in ALS. However, it should be noted that, due to the distal nature to the loci of lesion in the neuromuscular system, these measures reflect the integrated outcomes of many interrelated neurophysiological (e.g., motor unit recruitment and firing patterns), biomechanical (e.g., stiffness, viscosity), and behavioral (e.g., compensatory strategy) factors that underlie speech production. These factors remain hard to disentangle from lower-level measures as obtained by acoustic and kinematic techniques.

Surface electromyography (sEMG)—a noninvasive electrophysiological technique widely available in neurology practices—provides a more proximal and direct means of assessing neuromuscular pathology. While the current clinical applications of sEMG are qualitative and observational in nature, the rapid digital innovations over recent decades have given rise to powerful signal processing and data analytic techniques, allowing for better exploitation of the quantitative potential of sEMG. Along this line, Rong and colleagues have carried out a series of studies to develop fit-for-purpose facial sEMG analyses to quantitatively assess and characterize bulbar neuromuscular performance (43–45). Various features representing the amplitude, complexity, and regularity of jaw myoelectric activities, and the functional connectivity of the jaw muscle network have been identified and demonstrated sensitivity for detecting subclinical bulbar neuromuscular changes in ALS.

Thus far, a variety of objective measures have been obtained from various modalities (e.g., acoustic, kinematic, sEMG) to characterize and assess different constructs of bulbar/speech motor control (e.g., prosody, rhythm, functional connectivity). Mechanistically, these constructs are underpinned by a variety of neuromotor and physiological factors, which emerge from different levels of the bulbar motor system and are (differentially) susceptible to ALS. Some constructs such as prosody only emerge as a meaningful entity at the lower level of the bulbar motor system and is directly related to the behavioral end product (i.e., speech output). Other constructs such as functional connectivity are more relevant to the higher-level neuromuscular control, which underlies the modular organization of the bulbar motor system (i.e., organization of discreate bulbar muscles into holistic functional modules to facilitate task implementation). In addition, there are also constructs (e.g., rhythm) which constitute meaningful descriptors of the neuromotor and physiological processes at all levels. Given the complex nature of these constructs, measures obtained from a single modality as in most existing studies are unlikely to capture the disease effects on all facets of these constructs. To comprehensively assess these disease effects urges a multimodal measurement tool to hierarchically detect and quantify changes in all constructs at all applicable levels. Such a hierarchical multimodal measurement tool is currently lacking.

**Figure 1:**
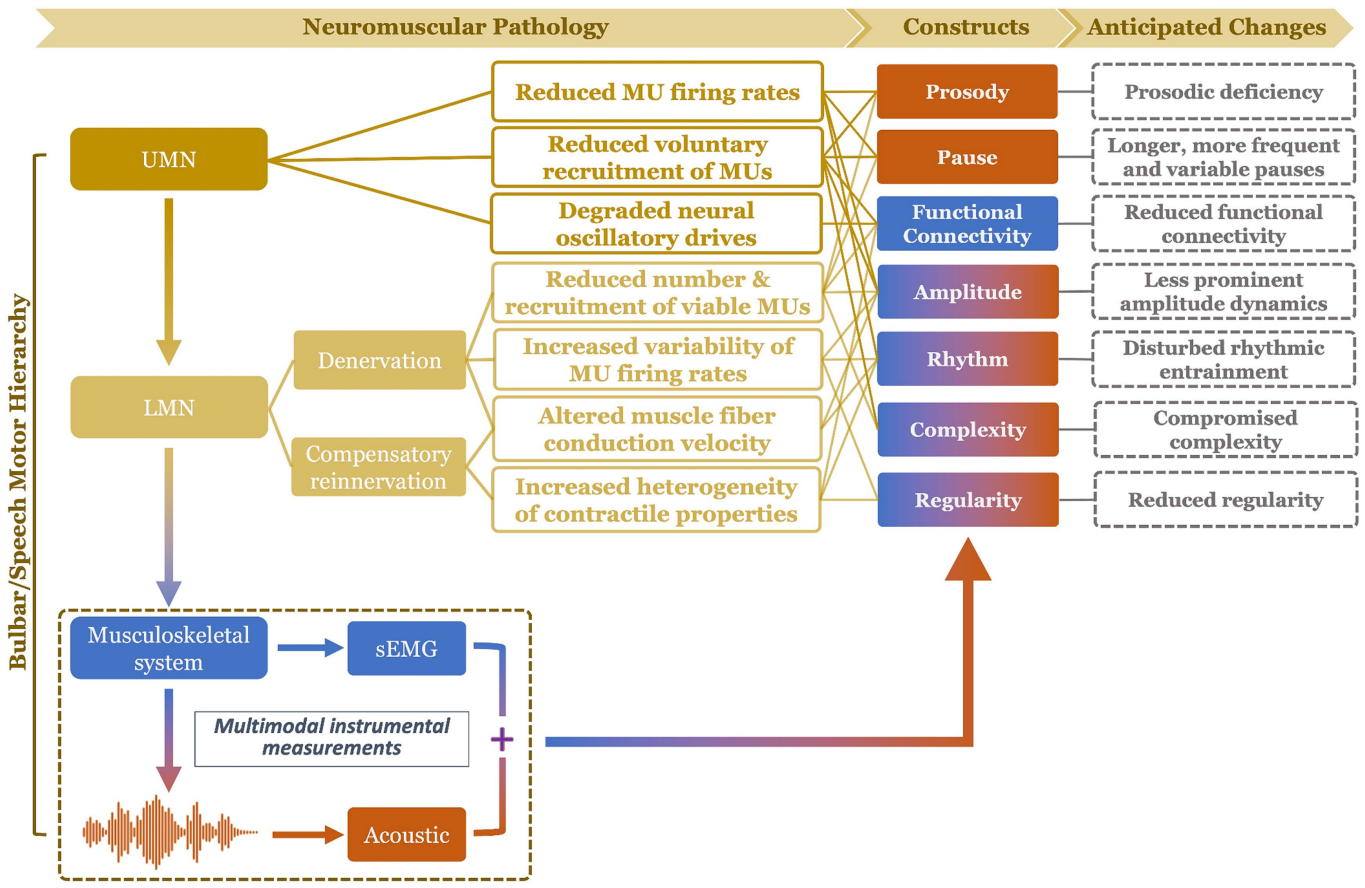
Schematic illustration of mechanistic links of the targeted constructs (and their anticipated changes) to bulbar neuromuscular pathology in amyotrophic lateral sclerosis. The color of the constructs reflects the modality of measurement: blue (functional connectivity) = surface electromyography (sEMG); orange (prosody, pause) = acoustic; mixed (amplitude, rhythm, complexity, regularity) = sEMG + acoustic. UMN, upper motor neuron; LMN, lower motor neuron; MU, motor unit.

To fill the above gap, this study proposes a multimodal measurement tool integrating two noninvasive instrumental techniques—facial sEMG and acoustic—with novel data analytics, to provide a hierarchical assessment of seven carefully selected constructs of bulbar/speech motor control at two distinct levels (i.e., neuromuscular, acoustic). Compared to other instrumental techniques, sEMG and acoustic instruments are readily available in neurology/speech clinics, rendering high clinical scalability to the proposed multimodal approach. The selected constructs, including prosody, pause, functional connectivity, amplitude, rhythm, complexity, and regularity, are mechanically and clinically relevant to bulbar involvement in ALS. The clinical relevance of these constructs has been well demonstrated by prior unimodal investigations as outlined above. Mechanistically, all constructs can be explanatorily linked to the combined effects of both UMN and LMN involvement on bulbar neuromuscular pathology. An overview of such explanatory links is provided in Figure 1 and further elaborated below.

UMN involvement is known to reduce the voluntary recruitment and firing rates of motor units (46). In addition, impairment of the motor cortical network due to UMN degeneration compromises the oscillatory drives which provide common neural inputs to comodulate functionally related bulbar muscles (47, 48). LMN involvement, on the other hand, results in denervation, which reduces the number and recruitment of viable motor units, increases the variability of motor unit firing rates, and changes the muscle fiber conduction velocity (49). As a compensatory response to denervation, collateral sprouting occurs spontaneously to reinnervate denervated muscle fibers by surviving axons at greater distances (50, 51). Yet, due to the variability in axon length, myelination, and safe factor, the contractile properties of the reinnervated and intact motor units tend to vary, resulting in more heterogeneous motor units discharge patterns.

Among all UMN and LMN-related neuromuscular changes, reduced voluntary recruitment, firing rates, and number of viable laryngeal and respiratory motor units would contribute to prosodic deficiency (e.g., monopitch) and abnormal pause (e.g., longer, more frequent and variable pauses between speech events) (31, 52, 53); such changes in prosody and pause can be detected from lower-level acoustic measurements. Reduced voluntary recruitment and number of viable bulbar motor units, degradation of oscillatory drives to comodulate the recruited motor units, and increased heterogeneity of the motor unit contractile properties tend to globally weaken the neural binding of bulbar muscles and in turn reduce the functional connectivity of the bulbar muscle network (43, 44, 46, 54–57); such a decrease in functional connectivity would be manifested by higher-level neuromuscular measurements as obtained by sEMG. Moreover, the global reduction of voluntary recruitment, firing rate, and number of viable bulbar motor units, as well as changes in firing rate variability and muscle fiber conduction velocity are expected to modify the amplitude dynamics of the bulbar motor system’s outputs at both higher (e.g., neuromuscular) and lower (e.g., acoustic) levels (46, 49, 58), as encoded across modalities. In addition, reduced voluntary recruitment and number of viable bulbar motor units can also compromise the ability of the bulbar motor system in conveying complex motor commands, which would be manifested by the complexity of the system’s outputs at all levels (10, 45). Abnormal discharge patterns of bulbar motor units, as relating to slower and more variable firing rates, more heterogeneous contractile properties, and changes in muscle fiber conduction velocity, can disrupt the rhythms of bulbar motor activities across modalities (49, 58–61). Lastly, increased variability and heterogeneity of the firing rates and contractile properties of bulbar motor units tend to globally reduce the regularity of bulbar motor activities across modalities (10, 45). Taken together, these evidenced-based explanatory links comprise a conceptual framework, illustrating the mechanistic relevance of the selected seven constructs to bulbar neuromuscular pathology in ALS.

This study aimed to (1) develop a multimodal measurement tool, integrating facial sEMG and acoustic instrumental techniques with a custom-developed, fully automated data analytic approach, to hierarchically assess and measure the seven targeted constructs during a speech task; (2) provide an initial validation of this measurement tool following the consensus-based V3 framework (62), focusing on the reliability, validity, and efficacy for early detection and progress monitoring of bulbar involvement in ALS. A speech task was selected because, as a fine oromotor behavior that requires sophisticated coordination of a variety of bulbar muscles, speech has been consistently demonstrated by prior studies to be sensitive for detecting early subclinical bulbar involvement in ALS (40, 41, 63, 64). Based on the theoretical and empirical evidence obtained from the conceptual framework in Figure 1 and prior unimodal investigations, we hypothesized that the multimodal measurement tool would hold high reliability (internal consistency), validity (concurrent and construct), and efficacy for early detection of prodromal bulbar involvement and for monitoring of bulbar progression across the prodromal and symptomatic stages. Moreover, it was further hypothesized that the multimodal measurement tool would outperform its unimodal subcomponents as obtained from each constituent modality.

## 2 Materials and methods

The study protocol was part of a larger-scope project, which was approved by the Institutional Review Board of the university medical center. Written informed consent was obtained from all participants. All study procedures were non-invasive and involved minimal risk.

### 2.1 Participants

Twenty-three participants including 13 individuals with ALS (8 men, 5 women; age: 38–74 years) and 10 age-matched neurologically healthy controls (3 men, 7 women; age: 38–81 years) took part in this study. Participants with ALS were further divided into two subgroups: (1) a bulbar-symptomatic subgroup (ALS + B), consisting of 7 individuals with overt clinical bulbar symptoms and (2) a bulbar-prodromal subgroup (ALS **−** B), consisting of 6 individuals without overt bulbar symptoms. The presence of bulbar symptoms was determined by standard clinical examination procedures administered by the second author. These procedures included an oral mechanism examination, a dysarthria screening/examination, a functional speech assessment, as well as swallow-related, patient-reported outcome measures, screeners (i.e., 3 oz. water test) or clinical and/or instrumental assessments [i.e., video fluoroscopic swallow study (VFSS) or fiberoptic endoscopic evaluation of swallowing (FEES)]. The inclusionary criteria were (1) being diagnosed with definite or probable ALS as per the revised El Escorial Criteria (2) for the ALS group or reporting no known neurological diseases or injury for the healthy control group; (2) speaking American English as the first and primary language; (3) passed hearing screening at 1,000, 2,000, and 4,000 Hz at 30 dB in at least one ear; (4) possessing adequate cognitive function to understand instructions and perform tasks as per by standard cognitive screening procedures.

To allow for evaluation of the concurrent validity of the multimodal measurement tool, two functional speech metrics were obtained from the Sentence Intelligibility Test (SIT)—a well-established and widely used functional speech assessment (65)—to serve as the criteria to correlate with the novel measures. In this test, participants read 11 randomly generated sentences with varying lengths ranging from 5 to 15 words. Speech was digitally recorded at 22,050 Hz and later orthographically transcribed and timed by two naïve listeners. The listeners were undergraduate students in the speech-language pathology major, who qualified the following conditions: (1) speaking American English as the first and primary language; (2) reporting normal speech, language, hearing, and cognitive functions, and (3) being unfamiliar with either the stimuli or the speaker profiles. Based on the judgment of each listener, speech intelligibility and speaking rate were derived as the percentage of words correctly transcribed out of the total number of words and the number of words per minute (WPM), respectively. Inter-listener reliability has been previously verified in another study [intelligibility: *r = 0.86, inter-listener discrepancy =* 1.02%±1.44% ; rate: *r = 0.99, inter-listener discrepancy =* 3.15±2.03 WPM; see (40)]. Lastly, both measures were averaged across listeners, giving rise to two standard metrics to index the functional speech capacities of the participants. Between the two metrics, speech intelligibility is a well-established outcome measure of communication effectiveness; speaking rate currently serves as the clinical proxy for staging and guiding management of dysarthria in ALS (66, 67). The demographic, clinical, and functional characteristics of the participants are provided in Supplementary Table S1 and summarized in Table 1.

**Table 1:** Statistical summary of demographic, clinical, and functional characteristics of participants.

Participant characteristics	ALS (*N* = 13)	Control (*N* = 10)	ALS vs. control comparison
**Demographic**			
Women (*n*%)	38.46%	70.00%	$\chi^{2}$ =1.17,p=0.28
Age, years (M; SD)	59.54; 12.78	66.80; 13.02	$F\left(1,21\right)=1.80,p=0.19$
**Clinical**			
Disease onset (*n*%)	Bulbar: 30.77%	N/A	N/A
	Spinal: 69.23%		
Stage of bulbar involvement (*n*%)	Prodromal: 53.85%	N/A	N/A
	Symptomatic: 46.15%		
Days since diagnosis (M; SD)	362.15; 422.56	N/A	N/A
**Functional**			
ALSFRS-R: total (M; SD)	36.92; 6.87	N/A	N/A
ALSFRS-R: bulbar (M; SD)	10.08; 2.40	N/A	N/A
Speech intelligibility, % (M; SD)	87.34; 25.03	99.45; 0.56	$F\left(1,21\right)=2.32,p=0.14$
Speaking rate, WPM (M; SD)	135.02; 41.27	183.65; 23.02	$F\left(1,21\right)=11.14,p=0.003^{*}$

ALS, amyotrophic lateral sclerosis; Control, healthy controls; WPM, words per minute; ALSFRS-R, ALS Functional Rating Scale-Revised. Significant statistical effects are marked by asterisks.

### 2.2 Experimental procedures

Data collection took place in a quiet lab environment. Participants were seated in an armed chair, wearing a set of surface electrodes on multiple regions of their head and face (for myoelectric data acquisition) as well as a head-mounted microphone around their ears (for acoustic data acquisition). All participants read the Rainbow Passage at their habitual speaking rate and loudness. Rainbow Passage is a standard phonetically balanced reading passage, consisting of 19 sentences with varying lengths. It has been used in previous studies and proven to robustly capture jaw muscle activities (43, 44).

**Figure 2:**
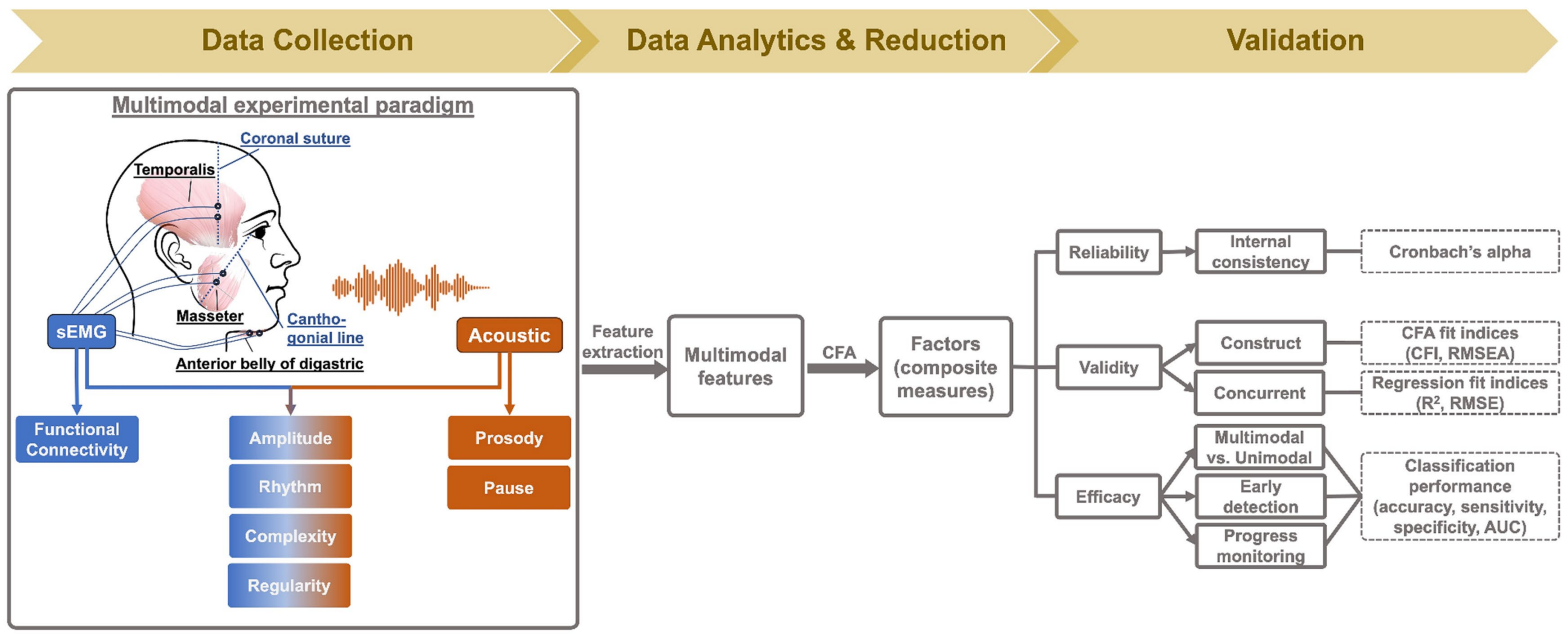
Methodology flowchart.

Myoelectric data were collected from three muscles of the mandibular system on the dominant side (right), including two jaw elevators—masseter and anterior temporalis—and one jaw depressor—anterior belly of digastric, using a wireless sEMG system (BIOPAC). Following the best practice guidelines for sEMG recording (68, 69), self-adhesive bipolar Ag/AgCl electrodes with 11 mm diameter size and 20 mm inter-electrode distance were attached to the skin over the belly of each target muscle, parallel to fiber orientation. Before placing the electrodes, the target areas of skin were prepared using an alcohol swab to increase skin conductance. To maximize reproducibility, several craniofacial anatomical landmarks were used to guide the placement of electrodes along the cantho-gonial line for masseter, vertically at the coronal suture for anterior temporalis, and submentally along the posterior-inferior direction for anterior belly of digastric. A ground electrode was attached to the participant’s shoulder. The placement of electrodes was then verified by calibration tasks such as jaw oscillations and clenching to ensure the jaw muscle activities were captured properly by the relevant electrodes. The analog signals were pre-amplified by 2,000 and band-passed filtered at 5–500 Hz by the wearable BioNormadix modules, digitized at 2,000 Hz by the MP160 data acquisition module, and finally recorded by the Acknowledge software. Acoustic data were acquired by a head-mounted microphone (DPA dfine 4188) placed approximately 5 cm away from the left lip corner, processed by the Behringer Xenyx 802 sound conditioner, and digitized and recorded at 22,050 Hz, simultaneously with the sEMG data. The flowchart in Figure 2 provides a graphic illustration of the experimental setup.

### 2.3 Data processing and feature extraction

Data analysis was conducted in MATLAB (R2021a), using a custom-developed, fully automated algorithmic program. To enhance data quality, sEMG recordings were pre-processed to remove electrical and mechanical artifacts, following the recommended procedures as used in prior work (45, 61, 70). Specifically, all sEMG channels were notch-filtered at 60 Hz and high-pass filtered at 20 Hz to remove power line noise and movement artifacts; DC offsets were then removed from each channel. Next, the pre-processed sEMG and acoustic signals were submitted to a feature extraction algorithm, which extracted 60 features from each sentence of the Rainbow Passage for each speaker (total N = 23 participants × 19 sentences = 437 speech samples). These features included (1) 3 acoustic-based measures of prosody, (2) 3 acoustic-based measures of pause, (3) 9 sEMG-based measures of functional connectivity, (4) 3 sEMG-based and 1 acoustic-based measures of amplitude, (5) 9 sEMG-based and 12 acoustic-based measures of rhythm, (6) 3 sEMG-based and 13 acoustic-based measures of complexity, and (7) 3 sEMG-based and 1 acoustic-based measures of regularity. An overview of these features is provided in Table 2, and the procedures for feature extraction are elaborated below.

**Table 2:** Summary of multimodal features.

Construct	Modality	Feature	Feature interpretation
Prosody	Acoustic	sdevF0.st	Standard deviation of f0 in semitone
		iqrF0.st	Interquartile range of f0 in semitone
		meanF0.st	Mean f0 in semitone
Pause	Acoustic	MeanDur_intrapause	Mean pause duration
		SdevDur_intrapause	Standard deviation of pause duration
		pct_intrapause	Percentage of pause time
Functional connectivity	sEMG	IMC_RTEMP_RMAS_theta_alpha	Theta/alpha-band intermuscular coherence (slow oscillatory drive)
		IMC_RTEMP_RABD_theta_alpha	
		IMC_RABD_RMAS_theta_alpha	
		IMC_RTEMP_RMAS_beta	Beta-band intermuscular coherence (fast oscillatory drive for submaximal tonic contractions)
		IMC_RTEMP_RABD_beta	
		IMC_RABD_RMAS_beta	
		IMC_RTEMP_RMAS_gamma	Low gamma-band intermuscular coherence (fast oscillatory drive for stronger tonic and phasic contractions)
		IMC_RTEMP_RABD_gamma	
		IMC_RABD_RMAS_gamma	
Amplitude	sEMG	density_RTEMP, density_RMAS, density_RABD	Density of myoelectric activities
	Acoustic	density_audio	Density of acoustic waveform
Rhythm	sEMG	mod_depth_theta_RTEMP	Envelope modulation depth for myoelectric activities at the theta timescale (syllable rhythm)
		mod_depth_theta_RMAS	
		mod_depth_theta_RABD	
		PSI_delta_theta_RTEMP	Delta-theta phase synchronization index for myoelectric activities (regularity of syllable stress)
		PSI_delta_theta_RMAS	
		PSI_delta_theta_RABD	
		PSI_theta_beta.gamma_RTEMP	Theta-beta/gamma phase synchronization index for myoelectric activities (temporal stability of syllable)
		PSI_theta_beta.gamma_RMAS	
		PSI_theta_beta.gamma_RABD	
	Acoustic	hbenvlp_mod_depth_theta_100_300	Envelope modulation depth for critical-band acoustic signals at the theta timescale (syllable rhythm)
		hbenvlp_mod_depth_theta_300_800	
		hbenvlp_mod_depth_theta_1000_3000	
		hbenvlp_mod_depth_theta_3000_8000	
		hbenvlp_PSI_delta_theta_100_300	Delta-theta phase synchronization index for critical-band acoustic signals (regularity of syllable stress)
		hbenvlp_PSI_delta_theta_300_800	
		hbenvlp_PSI_delta_theta_1000_3000	
		hbenvlp_PSI_delta_theta_3000_8000	
		hbenvlp_PSI_theta_beta.gamma_100_300	Theta-beta/gamma phase synchronization index for critical-band acoustic signals (temporal stability of syllable)
		hbenvlp_PSI_theta_beta.gamma_300_800	
		hbenvlp_PSI_theta_beta.gamma_1000_3000	
		hbenvlp_PSI_theta_beta.gamma_3000_8000	
Complexity	sEMG	DET_RTEMP, DET_RMAS, DET_RABD	Determinism of myoelectric activities
	Acoustic	DET_mfcc1 … DET_mfcc13	Determinism of MFCCs
Regularity	sEMG	ShanEn_RTEMP, ShanEn_RMAS, ShanEn_RABD	Shannon entropy of myoelectric activities
	Acoustic	ShanEn_audio	Shannon entropy of acoustic waveform

#### 2.3.1 Prosody

To extract prosodic features, the vocal fundamental frequency (f0) trace was obtained from the acoustic waveform, using the cross-correlation method (71). A moving window of 20-msec length for males and 10-msec length for females was applied to account for the pitch difference between male and female voice. Based on each f0 trace, the mean, standard deviation, and interquartile range were calculated on a semitone scale. These features provided objective metrics for assessing prosodic deficiency.

#### 2.3.2 Pause

The algorithm for pause analysis was adapted from the *Speech Pause Analysis (SPA)* program described in Green et al. (31). The differentiation between pauses and speech events is contingent on three threshold settings—minimum pause duration, minimum speech event duration, and minimum signal amplitude threshold. Green et al. (31) has tested a variety of threshold values in an ALS cohort with varying speech severities and shown that the measurements of pause can vary notably with threshold settings, especially for minimum pause duration and minimum signal amplitude threshold. Leveraging their findings with the current dataset (including both individuals with ALS and healthy controls), we selected the following baseline threshold values: minimum pause duration = 150 msec, minimum speech duration = 35 msec, minimum signal amplitude threshold = 0.04. These values roughly aligned with the pause-speech patterns of a speaker with an intermediate speaking rate at around 160 WPM. Given the large variability of speaking rate across speakers, we further scaled the baseline threshold value for minimum pause duration and minimum speech duration based on the ratio of the actual speaking rate to the baseline (i.e., 160 WPM) to account for the potential scaling effect related to the variability of speaking rate.

The scaled threshold values for minimum pause and speech durations along with the minimum signal amplitude threshold were entered into the algorithm to automatically identify all pauses and speech events from the acoustic waveform for each sentence of the passage. The mean and standard deviation of pause duration and the percentage of pause time were calculated for each sentence. These features allowed for assessment of abnormal pause (e.g., longer, more frequent and variable pauses) as previously reported in speakers with ALS (31).

#### 2.3.3 Functional connectivity

Functional connectivity was assessed by intermuscular coherence between all three pairs of muscles (temporalis-masseter, temporalis-digastric, masseter-digastric) in three frequency band: theta/alpha (4–12 Hz), beta (12–30 Hz), and low gamma (30–60 Hz), representing different oscillatory drives for comodulating these muscles. Specifically, the beta and low-gamma bands reflect fast oscillations originating directly from the motor cortical network. The two bands have slightly different functional roles: beta oscillations have been associated with submaximal tonic contractions, whereas low-gamma oscillations have been linked to attentionally more demanding, stronger tonic and phasic contractions (72–74). The theta/alpha band reflects slow oscillations related to diverse subcortical and cortical sources (e.g., hippocampus, brainstem) outside of the motor cortical network, enabling indirect motor control (75, 76). The feasibility of measuring intermuscular coherence in these bands from sEMG signals has been demonstrated by previous studies (44, 54, 56, 77–79).

Intermuscular coherence is defined as follows:


(1) $${IMC}_{xy}=\frac{{\left|S_{xy}\left(f\right)\right|}^{2}}{S_{xx}\left(f\right)S_{yy}\left(f\right)}$$

where ${IMC}_{xy}$ is intermuscular coherence, $S_{xy}\left(f\right)$ is the cross-spectrum between muscles, and $S_{xx}\left(f\right)$ and $S_{yy}\left(f\right)$ are the auto-spectra for the two muscles. To calculate intermuscular coherence, we followed similar procedures as in prior studies (44, 45). First, all sEMG signals were full wave rectified. Stationary 1-s epochs centered around the bursts were then identified and concatenated for each channel. The reconstructed sEMG data were fed into a coherence analysis to calculate the cross- and auto-spectra as specified in Eq. 1 for each pair of muscles, using a 4,096-point Fast Fourier Transform (FFT) applied over a sliding 1,024-point Hamming window with 75% overlap, following the recommendations by Terry and Griffin (80). In addition, a significance level corresponding to the upper 95% confidence limit under the hypothesis of independence between muscles was calculated as: $S=1-0.05^{1/\left(\hat{L}-1\right)}$ , where $\hat{L}$ is the adjusted number of overlapped segments (80, 81). Based on the coherence spectra, the mean coherence value in the theta/alpha, beta, and low-gamma bands were calculated for each muscle pair. Lastly, because only coherence above the significance level carries meaningful information about functional connectivity (or dependency), the theta/alpha, beta, and gamma-band coherence values were compared with the significant level, and all values below significance were set to zero. This last step eliminated arbitrary fluctuations in coherence measures that carried no meaningful functional information to improve the quality of the data.

#### 2.3.4 Amplitude

The measurement and quantification of amplitude has been a practically challenging task due to the variety of disease-unrelated contextual factors that can influence and confound the amplitude of motor speech outputs. For example, variations in mouth-to-microphone distance and audio gain would influence the amplitude of acoustic waveform; intrinsic inter-speaker variabilities in maximal force generation capacity would make a direct comparison of sEMG amplitude between speakers meaningless. Although these contextual factors can be controlled by proper calibration procedures, such procedures usually require additional equipment and/or tasks to be performed and cannot always be accommodated in a clinical setting. Since the goal of this study was to develop a clinically scalable bulbar measurement tool, a calibration-free method based on graph theoretical analysis was adopted to robustly measure and characterize the amplitude dynamics of both the acoustic waveform and the sEMG signals.

First, the original signal was transformed into a local standard deviation series as follows:


(2) $$V_{j}=\sqrt{\frac{\sum_{\left(j-1\right)*L+1}^{jL}{\left(U_{i}-{\bar{U}}_{j}\right)}^{2}}{L-1}}$$

where $\left[U_{j}\right]=\left[U_{1},U_{2},\ldots ,U_{N}\right]$ is the original signal, $\left[V_{j}\right]=\left[V_{1},V_{2},\ldots ,V_{M}\right]$ is the transformed local standard deviation series, and L is the length of intervals during which standard deviation is calculated. These intervals were set to be 50 msec long; thus, L was set to 1,103 ( 22,050Hz×50msec ) for acoustic signals and 100 ( 2,000Hz×50msec ) for sEMG signals.

Next, each local standard deviation series as derived by Eq. 2 was converted into a visibility graph, using similar procedure as in Melo et al. (82). In the graph, each point in $\left[V_{j}\right]$ was treated as a vertex; the connection between two vertices $V_{x}$ and $V_{y}$ was determined by the following criterion:


(3) $$\frac{V_{y}-V_{z}}{y-z}>\frac{V_{y}-V_{x}}{y-x}$$

where $V_{z}$ represents a vertex between $V_{x}$ and $V_{y}$ . If all $V_{z}$ meet the criterion in Eq. 3, $V_{x}$ and $V_{y}$ are regarding as being connected (i.e., visible to each other).

Lastly, a graph descriptor—density—was calculated to quantify the overall visibility level of the signal, using the following equation:


(4) $$\mathrm{density}=\frac{2\times m}{M\left(M-1\right)}$$

where M and m are the total number of vertices and edges of the graph, respectively. Density provides a robust means of characterizing the amplitude dynamics of the signal, which is not influenced by contextual factors such as inter-speaker variations in overall sound intensity level and force generation capacity. In a healthy speaker, the activation of the bulbar motor system related to speech production tends to generate distinctive amplitude dynamics in motor speech outputs (e.g., acoustic waveform, myoelectric activities). In a graphical term, such distinctiveness of amplitude dynamics is referred to as visibility; that is, vertices corresponding to highly activated states of the bulbar motor system tend to be more visible than those corresponding to less activated or inactive states. As such, reduced activation of the bulbar motor system in ALS is expected to decrease the overall visibility (as quantified by density using Eq. 4) of the graph.

#### 2.3.5 Rhythm

The rhythmic characteristics of both speech acoustic and sEMG activities were measured using an envelope modulation analysis previously developed by our research team. Technical details can be found in our prior studies (59, 60). Briefly here, this analysis characterizes the rhythmic modulation of speech events of different temporal granularities based on the envelope dynamics of motor speech signals at multiple timescales. In line with the rate of prosodic, syllabic, and sub-syllabic (onset-rime/phoneme) units in English, the delta (0.9–2.5 Hz), theta (2.5–12 Hz), and beta/gamma (12–40 Hz) timescales are used to characterize the rhythms of prosodic stress, syllables, and sub-syllabic segments, respectively. The rhythmic characteristics of these speech events are measured by the modulation depth at each timescale and the phase synchronization index (PSI) between timescales. Our prior work has shown that, of all rhythm metrics derived within and across the three timescales, ALS exhibits the most consistent effect on theta modulation depth and, to a lesser extent, on PSI between theta and its neighboring timescales (59, 60). Hence, this study focused on the three theta-related rhythm metrics. Specifically, theta modulation depth was used as a metric of syllable rhythm; delta-theta PSI, which measured the harmonic alignment of syllables within prosodic units, reflected the regularity of syllable stress; theta-beta/gamma PSI, which measured the harmonic alignment of sub-syllabic units (onset-time/phonemes) within syllables, provided a metric of temporal stability of syllable structures.

To measure the rhythmic characteristics of jaw muscle activities during speech, the non-speech intervals related to pauses were excluded from all sEMG channels based on the results of pause-speech differentiation as described in the pause analysis session. The Hilbert envelope of the reconstructed sEMG signals were derived and downsampled to 100 Hz. The power spectrum of each downsampled envelope was derived using 2048-point FFT with a hamming window. Theta modulation depth was calculated by summing the power at all frequency bins within the theta band and normalizing it by the total spectral power. To measure PSI, each downsampled envelope was further band-pass filtered in the time domain into three subcomponents (cutoffs of frequency: 0.9–2.5 Hz, 2.5–12 Hz, 12–40 Hz), using a fourth-order, zero-lag Butterworth filer. PSI was calculated between these subcomponents using the following equation:


(5) $$PSI=\;|e^{i\left(n\phi_{1}\left(t\right)-m\phi_{2}\left(t\right)\right)}|$$

where $\phi_{1}\left(t\right)$ and $\phi_{2}\left(t\right)$ are the instantaneous phase of the signals (i.e., subcomponents of the downsampled envelope) at time t ; n and m are integers reflecting the frequency relation between the two signals; $n\phi_{1}\left(t\right)-m\phi_{2}\left(t\right)$ represents the generalized phase difference. For delta-theta PSI and theta-beta/gamma PSI, the ratio of n : m was set to 2:1 and 3:1, respectively (83). All PSIs ranged between 0 and 1, with 0 denoting no synchrony and 1 reflecting perfect synchrony between signals.

To measure the rhythmic characteristics of speech acoustic activities, the acoustic waveform was reconstructed by excluding all pauses and concatenating all speech events. The reconstructed waveform was band-pass filtered in the spectral domain into 28 narrow spectral bands spanning between 100 and 10,000 Hz, matching the sound frequency representations on the cochlear map (84, 85). The Hilbert envelope of each narrow-band signal was calculated and downsampled to 100 Hz, giving rise to 28 narrow-band envelopes. Next, these narrow-band envelopes were combined into four critical-band envelopes within the following spectral frequency bands: 100–300 Hz, 300–800 Hz, 1,000–3,000 Hz, 3,000–8,000 Hz. These frequency bands are conventionally regarded as encoding the spectral contents of vocal pitch (f0), first formant (F1) of vowels, second formant (F2) of vowels, and noise energy associated with consonants. Lastly, the power spectra of all critical-band envelopes were derived, based on which the theta modulation depth was calculated for each critical-band envelope.

To calculate PSI, each narrow-band envelope was further band-pass filtered in the time domain into three envelope modulation series, using a fourth-order, zero-lag Butterworth filer with cutoffs that matched the delta (0.9–2.5 Hz), theta (2.5–12 Hz), and beta/gamma (12–40 Hz) rhythms. At each rhythm, all 28 narrow-band envelope modulation series were combined into four critical-band envelope modulation series. Delta-theta PSI and theta-beta/gamma PSI were calculated based on these critical-band envelope modulation series at the relevant timescales, using Eq. 5.

#### 2.3.6 Complexity

The complexity of both the acoustic and sEMG signals was measured using a nonlinear computational technique—Recurrence Quantification Analysis (RQA). RQA builds upon the recurrence plot, which is a visualization tool for displaying the dynamics of the phase space trajectory of a signal (86, 87). A recurrence plot consists of a varying number of recurrence points, each representing a state when the phase space trajectory returns to a previous state. To identify recurrence points requires specification of three technical parameters—embedding dimension m , time delay τ , and threshold ε . For technical details about these parameters, refer to Marwan (88). Based on the recurrence points identified, RQA derives a quantitative metric—determinism—as the ratio of recurrence points forming diagonal structures to all recurrence points. This metric quantifies the overall periodic content in the recurrence plot and is thus inversely related to the structural complexity of the signal.

To measure determinism of sEMG signals, the parameters were set to m=30 , τ=5 , ε=0.1 , as per both standard computational testing (e.g., false nearest neighbors and mutual information) and empirical evidence from our prior work (45). RQA was applied to non-overlapping 1-s segments; during these short segments, sEMG signals were considered as being relatively stable, and such stability was necessary for reliable detection of the underlying motor unit firing patterns. Determinism was calculated for each segment and then averaged across segments for each sEMG channel. To measure determinism of an acoustic signal, the first 13 mel-frequency cepstral coefficients (MFCCs) were computed, which encoded most of the speech-related acoustic content. These MFCCs were fed into RQA, with the parameters set to m=3 , τ=15 , ε=0.2 as determined per standard computational methods. Determinism was calculated for each MFCC.

#### 2.3.7 Regularity

To measure the regularity of both acoustic and sEMG signals, we combined wavelet packet decomposition (WPD) (89) with Shannon entropy, as previously applied to the analysis of unimodal data (45). Specifically, for each modality, the original series was decomposed into eight linear time-frequency representations, using 3-level WPD. Such decomposition was achieved through iterated low- and high-pass filtering, which has been demonstrated to be an effective means of delineating and characterizing physiological signals (90). For each time-frequency representation, Shannon entropy was calculated as:


(6) $$\mathrm{ShanEn}=-\underset{i}{\sum }{s_{i}}^{2}\log\left({s_{i}}^{2}\right)$$

where $s_{i}$ is the ith time-frequency representation. Lastly, Shannon entropy as computed using Eq. 6 was averaged across all eight time-frequency representations to provide an integrative metric of spectrotemporal irregularity for each signal (i.e., higher entropy corresponding to lower regularity).

### 2.4 Validation of the multimodal measurement tool

Validation of the multimodal measurement tool was carried out in the R statistical computing program (91). For all probability tests, both uncorrected ( α<0.05 ) and Bonferroni-corrected significance levels were considered for main effects, and the *p*-values were adjusted using the false discovery rate method for *post hoc* tests.

#### 2.4.1 Feature screening

To reduce the dimensionality of the feature set, a screening procedure was applied to exclude features with limited difference between the ALS and healthy control groups. To this end, the effect size of the between-group difference for all features was calculated using Cohen’s d. Of all features, those revealing at least a medium effect size ( $\left|d\right|>0.5$ ) were retained, and the rest were discarded. After the screening, the reduced feature set was submitted for validation testing in the following.

#### 2.4.2 Factorization (construct validity)

To evaluate construct validity, all features were normalized by z-score transformation and then fed into a 10-factor confirmatory factor analysis (CFA), using the *cfa* function with the maximum likelihood estimator in the *lavaan* package (92). The 10 factors aligned with the targeted constructs as measured by different instruments. Specifically, these factors included (1) prosody (*Pros_a*), (2) pause (*Pause_a*), (3) functional connectivity (*IMC_e*), (4) amplitude of acoustic waveform (*Amp_a*), (5) amplitude of myoelectric activity (*Amp_e*), (6) rhythm of acoustic activity (*Rhy_a*), (7) rhythm of myoelectric activity (*Rhy_e*), (8) complexity of acoustic activity (*Comp_a*), (9) complexity of myoelectric activity (*Comp_e*), and (10) regularity of myoelectric activity (*Reg_e*). Note that no factor was generated for the regularity of acoustic activity, because the feature related to this construct showed only a small between-group difference and was screened out in the last step (see more in the Results session).

During model fitting, all features were loaded onto the related factors (e.g., *meanF0.st, iqrF0.st, meanF0.st* were loaded onto *Pros_a*), generating a factor loading for each feature. By convention, a loading of at least 0.5 is required as an empirical cutoff for convergence (93). Based on this rule, all features with a loading lower than 0.5 were removed, and the remaining features were regarded as the component features of the factors. The CFA model generated a variety of statistics for evaluating how well the component features represented the targeted constructs. Of these statistics, we selected two most commonly used fit indices—comparative fit index (CFI) and root mean square error of approximation (RMSEA)—to evaluate the fit of the model. Lastly, the scores of all factors were calculated using the Bartlett method. These scores represented individual-level outcomes in the factor space.

#### 2.4.3 Internal consistency (reliability)

To evaluate reliability, we focused on the internal consistency of the multimodal measurement tool. Internal consistency reflects the homogeneity of all subparts of an instrument (94). To assess such homogeneity, we calculated the Cronbach’s alpha coefficient for all 10 factors of the multimodal measurement tool.

#### 2.4.4 Regression (concurrent validity)

To evaluate concurrent validity, the relationships between the factor scores of the multimodal measurements tool and two functional speech metrics—speech intelligibility and speaking rate—were assessed across all 437 speech samples using regression analysis. Three regression models were constructed for each outcome, including a multiple linear regression (MLR), which served as the benchmark for comparison with other models, and two nonlinear regressions based on the random forest (RF) and support vector machine (SVM) algorithms. RF is an ensemble machine learning (ML) algorithm that uses a combination of decision trees to solve classification or regression problems (95). RF has several strengths, including its robustness to data distribution and the built-in feature importance ranking to inform the relative contribution of each predictor to the outcome. SVM is another widely used ML algorithm, which allows for flexible mapping of the raw data into linear or nonlinear space using different kernels (96). In this study, the nonlinear radial basis function (RBF) kernel was selected due to the known precedence of subclinical bulbar involvement before functional speech decline in ALS, which would presumably give rise to a nonlinear relationship between the instrumental measures and functional speech metrics. The performance of all regression models was estimated through 5-fold cross-validation repeated 10 times and evaluated by two standard fit indices—R^2^ and root-mean-square error (RMSE).

#### 2.4.5 Classification (efficacy)

To compare the efficacy of the multimodal measurement tool with its the constituent unimodal subcomponents, ML classification models based on RF and SVM with the RBF kernel were utilized to differentiate between the ALS and healthy control samples, using different predictors. For the multimodal classification models, the scores of all 10 factors were entered as predictors; for the unimodal classification models, the scores of the subset of factors derived from each modality (*Pros_a, Pause_a, Amp_a, Rhy_a, Comp_a* for acoustic models; *IMC_e, Amp_e, Rhy_e, Comp_e, Reg_e* for sEMG models) were fed into the relevant models as predictors. The performance of all classification models was estimated through 5-fold cross-validation repeated 10 times and evaluated by the overall accuracy, sensitivity, and specificity of classification. In addition, the Receiver Operating Characteristic (ROC) curve and the area under the curve (AUC) were calculated for all classification models.

To further evaluate the efficacy of the multimodal measurement tool for early detection and progress monitoring of bulbar involvement in ALS, multiclass classification was utilized to differentiate between three classes—ALS + B, ALS − B, and healthy controls—using the scores of all 10 factors as predictors. Similar as above, both RF and SVM with the RBF kernel were used to fit the multiclass classification models. All models were cross-validated through 5-fold cross-validation repeated 10 times. Based on the cross-validation, the overall AUC and the accuracy, sensitivity, and specificity of classification between each two of the three classes were calculated. The efficacy for early detection and progress monitoring was indicated by the performance of classification between ALS − B and healthy controls and between ALS + B and ALS − B, respectively. To further evaluate the relevance of each predictor to early detection and progress monitoring, separate linear mixed effects (LME) models were applied to all factor scores (dependent variable), with a three-level categorical variable representing the subgrouping of participants (ALS + B, ALS − B, healthy control) as the fixed effect and a subject-dependent intercept as the random effect. Post-hoc pairwise comparisons were conducted based on estimated marginal means (*emmeans*) (97) and Cohen’s d to identify factors with significant, large differences for each comparison.

## 3 Results

### 3.1 Feature screening

**Figure 3:**
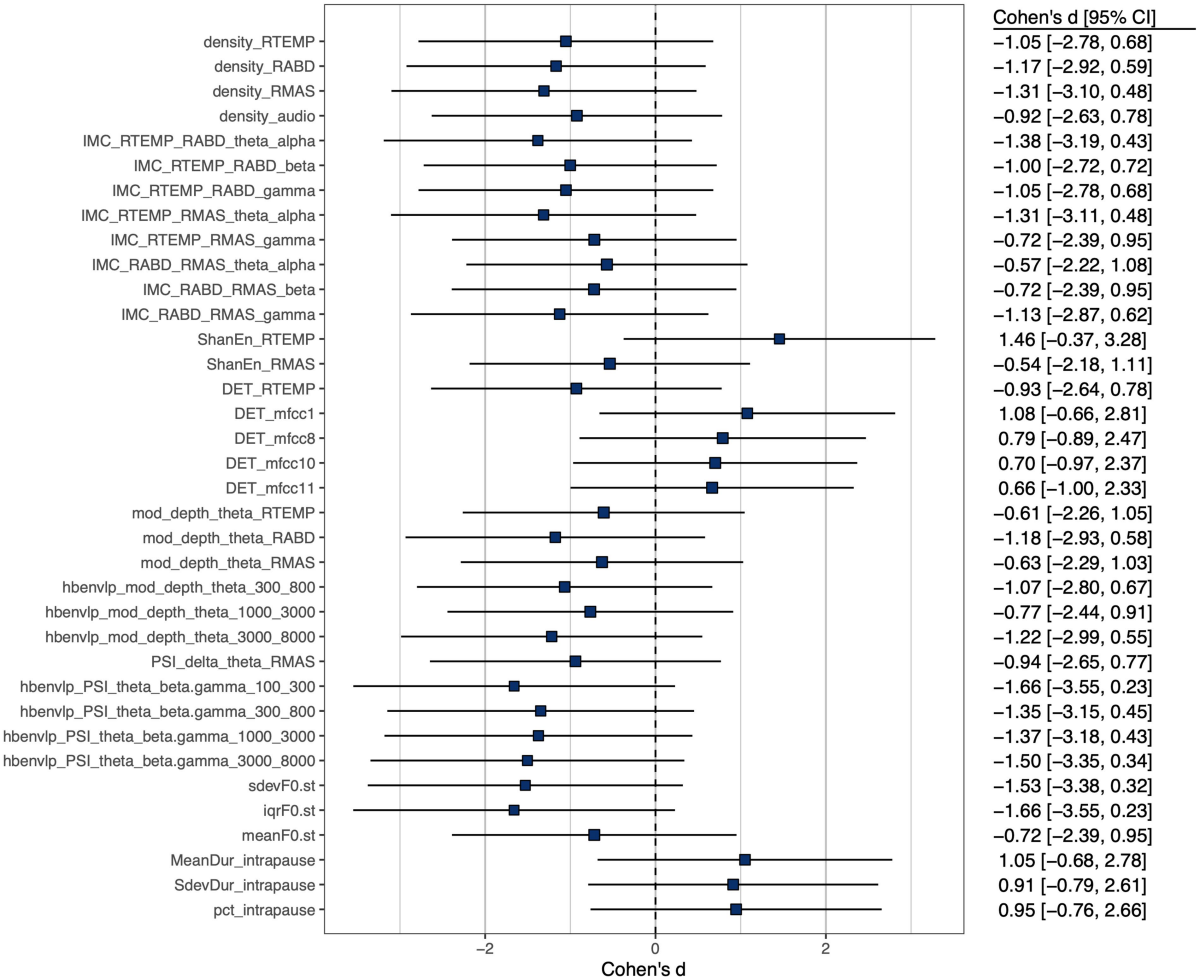
Cohen’s d effect size of between-group (ALS vs. healthy controls) difference of selected features, all exhibiting at least a medium effect size ( |d|>0.5 ).

A subset of features, including 3 prosodic features, 3 pause features, 8 features of functional connectivity, 3 sEMG-based amplitude features, 1 acoustic-based amplitude feature, 4 sEMG-based rhythm features, 7 acoustic-based rhythm features, 1 sEMG-based complexity feature, 4 acoustic-based complexity features, and 2 sEMG-based regularity features, were identified as having at least a medium effect size of the difference between the ALS and healthy control groups. Cohen’s d for these selected features is displayed graphically in Figure 3.

### 3.2 Factorization and construct validity

**Figure 4:**
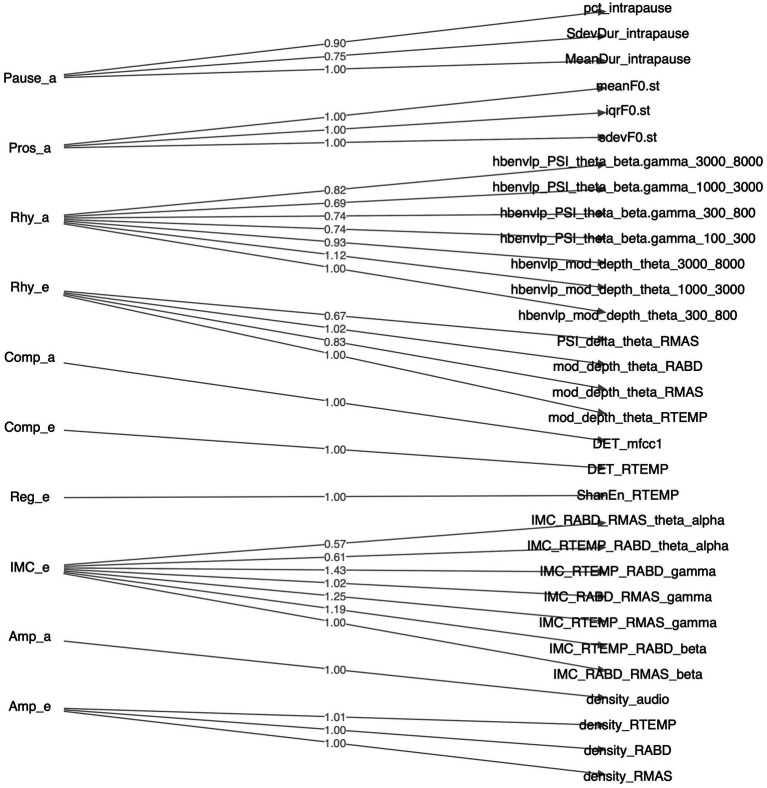
Structure of the factorial model constructed by confirmatory factor analysis. Factors, their component features, and the loadings of these features on the factors are displayed on the left, right, and middle sides of the diagram, respectively.

Of all 36 features as identified above, 31 loaded greater than 0.5 on the relevant factors; the remaining 5 features (1 for functional connectivity, 3 for acoustic-based complexity, 1 for sEMG-based regularity) had loadings smaller than 0.5 and were thus discarded. The structure of the resulting CFA model is depicted in Figure 4. The model fit indices are as follows: CFI=0.92 , RMSE=0.066 , which satisfy the conventional criteria for acceptable fit ( CFI>0.90 , RMSE<0.08 ) (98, 99), providing supportive evidence for the construct validity of the model.

### 3.3 Internal consistency

The internal consistency of the 10-factor CFA model was indicated by Cronbach’s alpha for all factors consisting of more than one component feature. Specifically, Cronbach’s alpha coefficient was 0.89 for *Pros_a*, 0.81 for *Pause_a*, 0.79 for *IMC_e*, 0.97 for *Amp_e*, 0.66 for *Rhy_e*, and 0.78 for *Rhy_a* (*Amp_a, Comp_e, Comp_a, Reg_e* only had one component feature, and Cronbach’s alpha was not applicable). While a consensus on the interpretation of Cronbach’s alpha is currently lacking, researchers have suggested a value >0.6 as satisfactory and a value >0.7 as ideal (93, 94). Because all Cronbach’s alpha coefficients calculated herein were greater than 0.6, with the majority being greater than 0.7, the internal consistency of the CFA model was regarding as satisfactory to ideal.

### 3.4 Concurrent validity

The statistics summary of the regression models for the relationships between the scores of all 10 factors and the two functional speech metrics (i.e., SIT-derived speech intelligibility and speaking rate) is provided in Table 3. For both outcomes, the RF and SVM-based nonlinear regression models showed overall better fit than the benchmark (i.e., MLR model), as indicated by smaller RMSE and greater R^2^. Moreover, regardless of ML algorithm, the nonlinear regression models consistently accounted for the majority of variance in both outcomes (79–88%), providing supportive evidence for the concurrent validity of the multimodal measurement tool. In addition to the overall fit, the importance of individual predictors to the outcomes, as indexed by decrease in node impurity, was estimated by the RF-based models. As shown in Table 3, *Rhy_a, IMC_e, Amp_e, Rhy_e,* and *Pause_a* were the top five predictors of speech intelligibility, whereas *Rhy_a, Comp_a, Rhy_e, Comp_e,* and *Pros_a* were the top five predictors of speaking rate.

**Table 3:** Statistics summary of regression models for the relationships between the factors obtained by the multimodal measurement tool (*Pros_a*, prosody; *Pause_a*, pause; *IMC_*e, functional connectivity; *Amp_e*, sEMG-based amplitude; *Amp_a*, acoustic-based amplitude; *Rhy_e*, sEMG-based rhythm; *Rhy_a*, acoustic-based rhythm; *Comp_e*, sEMG-based complexity; *Comp_a*, acoustic-based complexity; *Reg_e*, sEMG-based regularity) and the functional speech outcomes (speech intelligibility, speaking rate).

	Speech intelligibility			Speaking rate		
	RF	SVM	MLR	RF	SVM	MLR
**Overall fit**						
RMSE	7.51	6.48	14.31	17.71	18.55	23.76
R^2^	0.83	0.88	0.43	0.81	0.79	0.66
**Importance ranking**						
Pros_a	5378.42	N/A	N/A	45678.29	N/A	N/A
Pause_a	10614.18			32669.43		
IMC_e	35995.86			17689.22		
Amp_e	26814.74			17737.57		
Amp_a	1941.33			6076.06		
Rhy_e	16043.70			100002.67		
Rhy_a	38703.06			282729.37		
Comp_e	4080.26			54594.96		
Comp_a	7493.63			112659.34		
Reg_e	5379.34			31097.18		

sEMG, surface electromyohraphy; RF, random forest; SVM, support vector machine; MLR, multiple linear regression; RMSE, root-mean-square error. The importance of individual factors to the outcomes is measured by the total decrease in node impurity (i.e., residual sum of squares) derived by the RF algorithm; a larger value corresponds to greater importance.

### 3.5 Efficacy of classification between ALS and healthy controls: multimodal vs. unimodal

**Figure 5:**
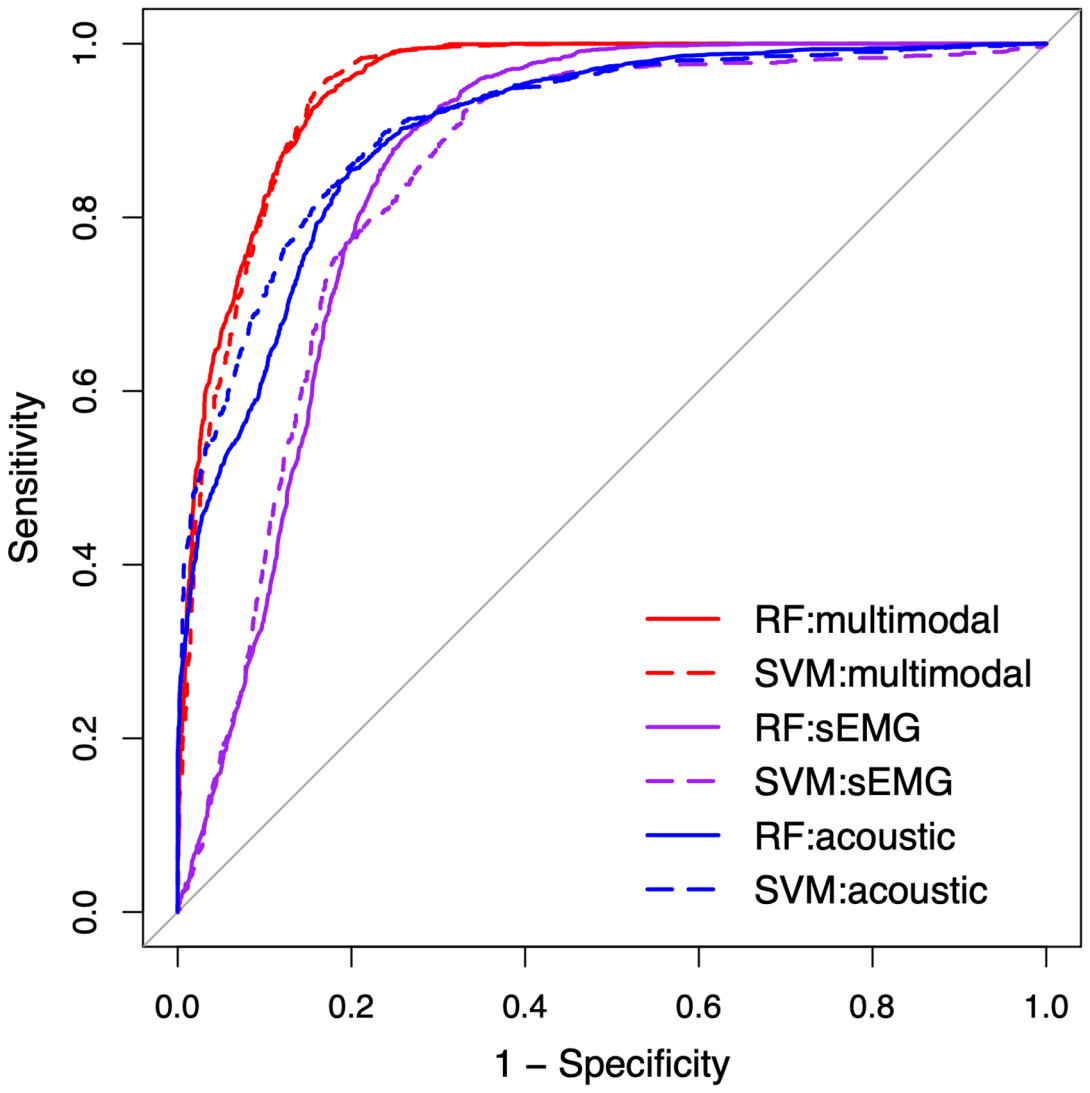
Receiver operating characteristic (ROC) curve for the classification models between the speech samples from individuals with amyotrophic lateral sclerosis (ALS) and healthy controls. RF, random forest; SVM, support vector machine; sEMG, surface electromyography.

Table 4 lists the performance metrics of all six classification models that used different predictors to differentiate between ALS and healthy control samples. The ROC curves for these models are displayed in Figure 5. The two ML algorithms revealed consistent performance on all metrics and ROC curve. Of all six models, the two based on multimodal predictors (i.e., scores of all 10 factors) showed the highest accuracy (0.88), sensitivity (0.88–0.89), specificity (0.88), and AUC (0.95), outperforming the other four models based on unimodal predictors (i.e., scores of sEMG or acoustic-based factors). These results confirmed the superior efficacy of the multimodal measurement tool over its unimodal subcomponents for detecting bulbar involvement in ALS.

**Table 4:** Performance of classification models that use different predictors (multimodal, sEMG, acoustic) to differentiate between the data samples for individuals with amyotrophic lateral sclerosis (ALS) and healthy controls.

	Multimodal		sEMG		Acoustic	
	RF	SVM	RF	SVM	RF	SVM
**Overall performance**						
Accuracy	0.88	0.88	0.80	0.80	0.82	0.83
Sensitivity	0.88	0.89	0.78	0.79	0.84	0.84
Specificity	0.88	0.88	0.81	0.80	0.79	0.81
AUC	0.95	0.95	0.86	0.85	0.90	0.90
**Importance ranking**						
Pros_a	75.36	N/A	N/A	N/A	89.07	N/A
Pause_a	12.48		N/A		36.56	
IMC_e	6.08		26.94		N/A	
Amp_e	3.79		29.56		N/A	
Amp_a	4.46		N/A		19.58	
Rhy_e	12.22		49.87		N/A	
Rhy_a	17.43		N/A		34.62	
Comp_e	24.73		46.91		N/A	
Comp_a	14.39		N/A		31.07	
Reg_e	39.90		57.60		N/A	

sEMG, surface electromyohraphy; RF, random forest; SVM, support vector machine; AUC, area under the curve; *Pros_a*, prosody; *Pause_a*, pause; *IMC_*e, functional connectivity; *Amp_e*, sEMG-based amplitude; *Amp_a*, acoustic-based amplitude; *Rhy_e*, sEMG-based rhythm; *Rhy_a*, acoustic-based rhythm; *Comp_e*, sEMG-based complexity; *Comp_a*, acoustic-based complexity; *Reg_e*, sEMG-based regularity. The importance of individual factors to the classification is measured by the total decrease in node impurity (i.e., Gini index) derived by the RF algorithm; a larger value corresponds to greater importance.

### 3.6 Efficacy for early detection and progress monitoring of bulbar involvement in ALS

The results of multiclass classification between ALS − B, ALS + B, and healthy controls are provided in Table 5. The two ML algorithms exhibited consistently good performance, both on the classification across all three classes ( AUC=0.96 ) and on the classification between each two of the three classes ( accuracy≥0.91 ). More specifically for the latter, the better classification model of the two (1) exhibited both high sensitivity (0.90) and specificity (0.96) for differentiating ALS + B from healthy controls, providing corroborating evidence for the efficacy of the multimodal measurement tool for detecting clinical confirmed bulbar involvement in ALS; (2) was highly specific (0.94) for differentiating ALS − B from healthy controls, confirming the specificity of the multimodal measurement tool for detecting prodromal bulbar involvement in ALS; (3) was both highly sensitive (0.94) and specific (0.98) for differentiating ALS + B from ALS − B, substantiating the efficacy of the multimodal measurement tool for monitoring the progression of bulbar involvement from prodromal to symptomatic stages.

**Table 5:** Multiclass classification between individuals at the prodromal stage of bulbar involvement secondary to amyotrophic lateral sclerosis (ALS − B), individuals at the symptomatic stage of bulbar involvement secondary to amyotrophic lateral sclerosis (ALS + B), and healthy controls (Control).

		ALS − B vs. control			ALS + B vs. ALS − B			ALS + B vs. control		
Model	Multiclass AUC	Acc.	Sens.	Spec.	Acc.	Sens.	Spec.	Acc.	Sens.	Spec.
RF	0.96	0.91	0.86	0.93	0.95	0.94	0.97	0.92	0.83	0.97
SVM	0.96	0.91	0.87	0.94	0.96	0.94	0.98	0.93	0.90	0.96

RF, random forest; SVM, support vector machine; AUC, area under the curve; Acc, accuracy; Sens, sensitivity; Spec, specificity.

The statistics summary of the LME models and Cohen’s d for factor score comparisons across the three classes are provided in Table 6. Among the 10 factors, a significant main effect of subgroup (ALS − B, ALS + B, healthy controls) was found on the scores of 8 factors ( p<0.05 ); two of these factors (*Amp_a, Rhy_a*) survived Bonferroni correction of significance level for multiple tests ( p<0.005 ). Based on the *post hoc* comparisons between ALS − B and healthy controls, *Pros_a* showed a significant large decrease; *Reg_e* showed a significant large increase; *Comp_e* exhibited a marginally significant large decrease. Based on the post-hoc comparisons between ALS + B and ALS − B, *Rhy_a* showed a significant large decrease; *Rhy_e* showed an insignificant large decrease; *Amp_a* revealed a significant medium decrease. For the post-hoc comparisons between ALS + B and healthy controls, all factors except *Comp_e* and *Pause_a* showed a significant difference, most with a large effect size.

**Table 6:** Statistical results for the main effect of subgroup (ALS − B: individuals at the prodromal stage of bulbar involvement secondary to amyotrophic lateral sclerosis; ALS + B: individuals at the symptomatic stage of bulbar involvement secondary to amyotrophic lateral sclerosis; Control: healthy controls) on the scores of the 10 factors and the *post hoc* pairwise comparisons.

Factor	Main effect				Pairwise comparisons						
			ALS − B vs. control			ALS + B vs. ALS − B			ALS + B vs. control		
	F	*p*	t	*p*	es	t	*p*	es	t	*p*	es
Amp_e	6.26	**0.0078**	−1.62	0.12	−0.40	−1.85	0.12	−0.52	−3.53	***0.0063***	***−0.89***
Comp_e	2.87	0.080	−2.39	0.080	***−1.18***	1.11	0.29	0.46	−1.08	0.29	−0.66
Rhy_e	6.81	**0.0056**	−1.78	0.091	−0.73	−1.83	0.091	***−0.82***	−3.68	***0.0045***	***−1.52***
IMC_e	6.04	**0.0090**	−1.44	0.17	−0.35	−1.95	0.098	−0.56	−3.47	***0.0072***	***−0.89***
Reg_e	5.65	**0.011**	2.40	***0.040***	***1.13***	0.76	0.46	0.28	3.11	***0.017***	***1.27***
Amp_a	7.18	***0.0045***	−0.57	0.58	−0.089	−2.90	***0.013***	−0.58	−3.67	***0.0045***	−0.66
Comp_a	3.72	**0.042**	1.50	0.22	0.62	1.16	0.26	0.50	2.68	***0.043***	***1.24***
Rhy_a	8.60	***0.0020***	−1.50	0.15	−0.50	−2.52	***0.031***	***−0.87***	−4.14	***0.0015***	***−1.32***
Pros_a	6.98	**0.0050**	−3.58	***0.0057***	***−1.93***	0.92	0.37	0.42	−2.41	***0.038***	***−1.14***
Pause_a	3.17	0.064	1.57	0.20	0.67	0.87	0.40	0.36	2.43	0.074	***1.04***

Significant main effects at the uncorrected significance level ( α<0.05 ) are shown in bold, and those surviving Bonferroni correction ( α<0.005 ) are further highlighted in bold-italic. Significant *p*-values and large effect sizes for the pairwise comparisons are shown in bold-italic. F, *F*-value; *p*, *p*-value; es, Cohen’s effect size. *p*-values for pairwise comparisons are adjusted by the false discovery rate method.

## 4 Discussion

This study developed and validated a multimodal measurement tool, which built upon two clinically readily available, noninvasive instrumental techniques (sEMG and acoustic) coupled with a fit-for-purpose, fully automated data analytic algorithm, aiming to offer an objective means of assessing bulbar involvement in ALS. Compared to the existing clinical and instrumental bulbar assessment methods, this tool is innovative and advantageous in that it provides a holistic assessment of seven carefully selected constructs of bulbar/speech motor control (i.e., prosody, pause, functional connectivity, amplitude, rhythm, complexity, regularity), which are mechanically linked to both UMN and LMN-related bulbar neuromuscular pathology and generate clinically interpretable outcomes that have both assessment and management implications.

Using the multimodal measurement tool, a variety of features were extracted from the sEMG and acoustic modalities during a speech task. These features were successfully clustered into 10 internally consistent, structurally valid factors in alignment with the targeted constructs as encoded in the sEMG or acoustic modality. Using machine learning algorithms, the scores of the 10 factors demonstrated (1) strong correlations with the functional speech outcomes ( $R^{2}=79-88\%$ ) and (2) high accuracy ( 0.91−0.96 ) for detecting subclinical bulbar changes, both during the clinically silent prodromal stage and during the transition from prodromal to symptomatic stages. These results together provide compelling initial evidence for the reliability and validity of the multimodal measurement tool and its efficacy for both early detection and progress monitoring of bulbar involvement in ALS. Moreover, the multimodal measurement tool outperformed its unimodal subcomponents as obtained from each constituent modality for detecting bulbar involvement, which paves the way for combining multimodal instrumental measurements in future bulbar assessment.

### 4.1 Mechanistic links between the outcomes of the multimodal measurement tool and bulbar neuromuscular pathology in ALS

A major strength of the multimodal measurement tool in this study is the mechanistic relevance of the constructs being measured to the neuromuscular pathology of bulbar involvement in ALS. Such mechanistic relevance, as conceptualized in Figure 1, is empirically supported by the factorial model (Figure 4) and the observed disease effects on the component features of this model (Figure 3). Specifically, the successful factorization of all features into composite outcomes (factors) in alignment with the theoretical constructs demonstrates the structural construct validity of these features in providing true representations of the targeted constructs. Moreover, the internal consistency of the outcomes, as indicated by Cronbach’s alpha ( >0.6 ), further provides evidence for the homogeneity of the features in measuring the targeted constructs.

Importantly, the component features of the factorial model reveal disease-related changes in expected ways consistent with the neuromuscular pathology of bulbar involvement in ALS. Based on the effect sizes in Figure 3, all prosodic features show large decreases, implying prosodic deficiency. All pause measures show large increases, revealing a trend toward longer, more frequent and variable pauses between speech events. This trend is in agreement with the previously reported abnormal pause pattern of speakers with ALS (31, 64, 100, 101). Intermuscular coherence exhibits medium-to-large decreases across all frequency bands (theta/alpha, beta, gamma), pointing toward a global trend of reduced functional connectivity of the mandibular muscle network. Among the three bands, reduced beta-band coherence between jaw muscles has been previously reported (44, 45), while the current study further extends the finding to the coherence in two additional bands. Taken together, these results suggest that reduced functional connectivity of the mandibular muscle network is associated with the impairment of multiple oscillatory drives from both motor cortical and other related cortical and subcortical sources.

Regarding amplitude density, both sEMG and acoustic signals exhibit large decreases, reflecting less distinctive amplitude dynamics of myoelectric and acoustic activities. Regarding rhythm, the theta modulation depth for all jaw muscles and the critical-band envelopes in the 300–800, 1,000–3,000, and 3,000–8,000 Hz bands show medium-to-large decreases, providing converging evidence for reduced entrainment of myoelectric and acoustic-physiological activities to syllable rhythm, especially pertaining to the articulation of vowels (as encoded in the 300–800 and 1,000–3,000 Hz bands) and consonants (as encoded in the 3,000–8,000 Hz band). Moreover, the delta-theta PSI for masseter shows a large decrease, reflecting reduced harmonic entrainment of masseter to produce syllable stress; additionally, the theta-beta/gamma PSI for all critical-band envelopes exhibit large decreases, revealing reduced harmonic entrainment of acoustic-physiological activities to produce stable temporal structures of syllables. Together, the observed rhythmic disturbances within and across timescales are in keeping with prior finding of impaired entrainment of motor speech activities to the rhythms of the underlying linguistic events (59, 60).

Regarding complexity, the determinism of temporalis activity exhibits a large decrease, which corresponds to increased complexity. This increase, although not in entire agreement with the expected pathological change in complexity as conceptualized in Figure 1, resonates with the finding of prior empirical research (45). Instead of being a direct pathological consequence, such an increase in the complexity of temporalis activity has been interpreted as a secondary functional adaptation (45). This adaptation is characterized by reduced functional recruitment of masseter—a preferred agonist for speech-related jaw elevation in healthy speakers (102)—and an accompanying increase in the recruitment of temporalis, which is a biomechanically less advantageous jaw agonist for speech production but is histochemically more resistant to ALS than masseter due to the higher composition of slow fibers (55, 57, 103). The resulting increase in functional recruitment of temporalis could yield more complex activation patterns. In contrast, the determinism of the first-order MFCC shows a large increase, reflecting less complex variations in the spectral shape of the source-filter transfer function during running speech. Such a change would conceivably compromise the ability of the speech production system in conveying complex linguistic information, in turn degrading the outcome of functional communication. Regarding regularity, a large increase in Shannon entropy is found in temporalis, reflecting reduced regularity of its myoelectric activity. Consistently, prior work has reported a similar decrease in the regularity of jaw myoelectric activities, especially of temporalis (45).

In summary, the features extracted by the multimodal measurement tool are intrinsically homogeneous and valid in representing the targeted theoretical constructs of bulbar/speech motor control. The disease-related changes in all features are interpretable in such a way as to align with the conceptualized effects of combined UMN and LMN involvement on the targeted constructs. These findings empirically substantiate the mechanistic links between the outcomes of the multimodal measurement tool and bulbar neuromuscular pathology, which are of fundamental importance for a comprehensive understanding and targeted assessment of bulbar involvement in ALS.

### 4.2 Relation of the multimodal measurement tool to the existing clinical and functional criteria for diagnosing and evaluating bulbar involvement in ALS

Based on the nonlinear ML regressions, the outcomes of the multimodal measurement tool (i.e., scores of the 10 factors) demonstrate strong correlations with the two functional speech metrics ( $R^{2}=0.79-0.88$ ; see Table 3), providing evidence for the concurrent validity of the multimodal measurement tool. Linear regression shows overall worse fit ( $R^{2}=0.43-0.66$ ), especially for the intelligibility model. This finding is not surprising given the known nonlinearity of intelligibility decline at an incremental rate over the time course of bulbar involvement in ALS (64, 101). Speaking rate, on the other hand, declines more rapidly, especially during the early stage, resulting in a more linear decline pattern than that of intelligibility (66, 101).

Based on the importance rankings, the 10 factors exhibit differential contributions to the functional speech metrics. Notably, the factors related to rhythm (*Rhy_a, Rhy_e*) are among the top contributors to both intelligibility and speaking rate, highlighting the functional importance of rhythm in conveying intelligible linguistic information at an appropriate rate. Such functional importance can be attributed to an oscillation-based speech processing mechanism known as rhythmic entrainment. On the perception side, prior neuroacoustic studies have associated such a mechanism with a bottom-up process through phase-locking of theta oscillations in the auditory cortex to the rise-fall dynamics of syllables, allowing the incoming sound stream to be parsed into discrete syllables (104–106). Such syllable parsing further provides a gate-keeping mechanism to entrain oscillations at other timescales (e.g., delta, beta/gamma) for hierarchical multiscale speech processing (107). On the production side, behavioral studies have provided parallel evidence showing entrainment of the motor speech system to the theta rhythm (108, 109). Linking the processes on the production and perception sides reveals a common paradigm of entrainment centered on the theta (syllable) rhythm. Such an entrainment paradigm, in the context of the current study, corroborates with the strong correlations between the theta-related rhythm metrics of motor speech activities and the functional speech outcomes. From a clinical perspective, this finding provides an impetus to explore the role of syllable rhythm in the management of dysarthria in ALS.

In addition to the correlations with the functional speech outcomes, the factors also show high efficacy for differentiating ALS + B from healthy controls (see Table 5). This finding demonstrates the clinical validity of the multimodal measurement tool for detecting bulbar involvement as confirmed by the current diagnostic criteria. Taken together, the results of the regression and classification analyses provide converging evidence for the validity of the multimodal measurement tool in relation to the existing clinical and functional criteria for diagnosing and evaluating bulbar involvement in ALS.

### 4.3 Combining multimodal instrumental techniques to improve the early detection and monitoring of bulbar involvement in ALS: a new promising direction

As shown in Table 4 and Figure 5, the multimodal measurement tool as a whole outperforms its unimodal subcomponents for differentiating between ALS and healthy control samples. It is thus conceivable that the measures obtained by sEMG and acoustic techniques provide complementary information to the extent that leads to improved detection of bulbar involvement through combining the two modalities. Moreover, the top five predictors of the classification model include both sEMG-based (*Reg_e, Comp_e*) and acoustic-based (*Pros_a, Rhy_a, Comp_a*) measures, providing further evidence for the need for both modalities to achieve satisfactory classification performance. Interestingly, four of these predictors (*Pros_a, Comp_e, Rhy_a, Comp_a*) overlap with the top contributors to speaking rate—a metric currently used by clinicians to stage the course of dysarthria progression in ALS patients (66, 110). This observation accentuates the critical roles of prosody, complexity, and rhythm, as measured crossmodally, in both the detection and staging of bulbar/speech impairment. According to these findings, the multimodal measurement tool demonstrates a clear strength over its unimodal subcomponents, paving the way for combining multimodal instrumental techniques as a means to improve the efficacy of bulbar assessment in future clinical practice.

Based on the results of multiclass classification as shown in Table 5, the multimodal measurement tool shows promise for both early detection of prodromal bulbar involvement and for monitoring of bulbar progression from prodromal to symptomatic stages. Regarding early detection, the 10 factors combined achieve 91% accuracy in differentiating ALS − B from healthy controls. Of all factors, prosody (*Pros_a*), regularity of temporalis activity (*Reg_e*), and complexity of temporalis activity (*Comp_e*) exhibit the greatest changes between ALS − B and healthy controls (see Table 6). Regarding progress monitoring, the 10 factors combined are over 93% accurate in differentiating ALS + B from ALS − B. Of all factors, the rhythm of both acoustic and myoelectric activities (*Rhy_a, Rhy_e*) reveal the greatest changes between the two subgroups of patients.

The above findings elucidate differential susceptibility of the 10 factors to different stages of bulbar involvement in ALS, rendering them potential for distinct clinical applications. Specifically, prosody and regularity are most susceptible to the prodromal stage of bulbar involvement, and their declines slow down or stabilize as the disease progresses from prodromal to symptomatic stages. Complexity is also susceptible to early prodromal bulbar involvement, but its change is reversed during the transition from prodromal to symptomatic stages such that the earlier-observed disease effect is lost during the symptomatic stage. This observation is most likely associated with the nature of the early-stage increase in the complexity of temporalis activity, which is presumed to reflect a secondary functional adaptation rather than a direct pathological consequence. Such an adaptation may become more difficult as trigeminal motor neurons continue to degenerate with disease progression, leading to the ultimate loss of the adaptation during a later stage. Despite the differential responses to bulbar progression, prosody, regularity, and complexity all exhibit large detectible changes during the prodromal stage, making them good candidates for early markers of bulbar involvement to improve the detection of prodromal changes in different constructs of bulbar/speech motor control. On the other hand, rhythm is less susceptible to early prodromal bulbar involvement but exhibits an accelerated decline during the transition from prodromal to symptomatic stages. Given the high responsiveness to this transition, the factors related to acoustic and sEMG rhythms may be good candidates for progression markers to monitor bulbar involvement in ALS. For the rest of the factors (e.g., *IMC_e, Amp_e, Amp_a, Comp_a, Pause_a*), most exhibit a slower but steady decline over the entire course of bulbar involvement. The disease effects on these factors are only modest during the prodromal stage yet accumulate over time, eventually culminating in notable changes in the factor scores when the disease progresses to the symptomatic stage. These factors are less preferable as markers for early detection or progress monitoring but still contain important explanatory information about bulbar dysfunction in ALS.

### 4.4 Clinical implications

The novel multimodal measurement tool in this study has several advantages that render it highly scalable for clinical applications. The major advantages include (1) use of clinically readily available, noninvasive instrumental techniques, (2) standardization of instrumentation and setup (e.g., electrode placement and orientation with reference to anatomical landmarks) to maximize inter-subject and intersession reliability, (3) standardization and objectification of outcome measures to allow for unbiased interpretation and report of results, and (4) nonexpert-friendliness, owing to the fully automated data processing and analysis methods. Moreover, based on an informal post-study survey, all participants reported having no significant physical or mental discomfort related to the study procedures and were willing to come back for a follow-up session provided that they had the functional capacity to do so.

The multimodal measurement tool has three clinical implications. First, it can facilitate early access to optimal clinical management of bulbar dysfunction, especially pertaining to voice preservation. Regardless of disease onset, early consultation on voice preservation is advocated, which allows patients to record and bank their voice for future use on an AAC device when speech is no longer an effective means of communication. Given the demonstrated efficacy for detecting prodromal bulbar changes, the multimodal measurement tool may improve the early diagnosis of bulbar involvement, which would allow patients more time to consult with clinicians and bank their voice before their verbal communication starts to deteriorate. Second, the multimodal measurement tool can improve stage-dependent management of dysarthria. The current clinical guideline for dysarthria management follows a staging principle, which defines five stages of intervention focusing, respectively, on information gathering, environmental modification (e.g., minimizing background noise), behavioral modification (e.g., volitional speaking rate reduction, hyper-articulation), introduction of AAC as a primary or supplementary means of communication, and full reliance on AAC (110). To effectively implement such stage-dependent management, it is most important to identify critical periods when the patient’s communication capacity is expected to change, in order to allow for timely adjustment of intervention to meet the new communication needs. Given the demonstrated responsiveness to bulbar changes across stages, the multimodal measurement tool may provide a monitoring technique to track changes related to bulbar progression; based on these changes, critical periods can be identified to assist clinicians in implementing the staging principle of intervention to target the evolving communication needs of patients throughout their disease course.

Lastly, the multimodal measurement tool may provide a novel means for deep phenotyping of speech impairment in individuals with ALS. As a hallmark of ALS, heterogeneity is well recognized across functional domains including speech. The presentation and progression of speech impairment vary substantially across individuals. To delineate such heterogeneity requires deep phenotyping of the neuromotor and physiological underpinnings of speech impairment rather than clinical manifestations alone. The multimodal measurement tool in this study provides such a means for deep speech phenotyping. The individual-level phenotype data generated by this tool can guide the selection and stratification of participants for clinical trials to facilitate the discovery of new treatments. In addition, the multimodal measurement tool can also be used to collect phenotype data during the course of intervention/treatment to provide insights into the intervention/treatment progress. Such insights can guide clinicians in selecting and adapting evidence-based practice elements to the responses of individual patients during the intervention/treatment course, which would ultimately shape a new path for measurement-based care—a practice of basing clinical care on patient data collected throughout treatment/intervention (111).

### 4.5 Limitations and future directions

Despite the promising findings of this study, it must be acknowledged that these findings were based on a relatively small sample collected from a single site. Further larger-scale cross-site validation is warranted to substantiate the reliability, validity, and efficacy of the multimodal measurement tool in future work. Along these lines, the current study served as a starting point and was designed in a way to facilitate future efforts. Specifically, the study protocol was developed and implemented in a way as to maximize reproducibility and replication, through standardization of experimental procedures (e.g., electrodes placement and orientation) and automation of data processing and analysis. Validation testing was conducted on (1) a heterogeneous ALS cohort, which provided a representative sample of the entire course of bulbar involvement spanning prodromal to symptomatic stages, and (2) a phonetically balanced speech task consisting of 19 sentences with varying contexts and lengths, which individually rendered sufficient variability and together provided a comprehensive coverage of the phonetic inventory to ensure the robustness and generalizability of the results.

It should also be noted that this study was cross-sectional in nature. Thus, the efficacy of the multimodal measurement tool for progress monitoring was evaluated based on the comparison of cross-sectional data obtained from individuals at different stages of bulbar involvement (ALS − B, ALS + B) rather than longitudinal data reflecting the progression of bulbar involvement within an individual. The between-subject differences in the former were by no means equivalent to the within-subject differences in the latter. Future work should focus on further testing the responsiveness of the multimodal measurement tool to the progression of bulbar involvement within an individual based on longitudinal data.

There are several factors that can potentially bias or impact the generalization of the results and should be further delineated in future research. First, while we considered the participants without overt clinical bulbar symptoms (i.e., ALS − B) as being at the prodromal stage of bulbar involvement (i.e., “false negatives”), it should be pointed out that population-wise there is a small proportion of patients with ALS who never develop bulbar involvement throughout their disease course (i.e., “true negatives”). In our study, the mean disease duration of the ALS − B subgroup is 453 days post-diagnosis, which according to a prior longitudinal study renders 50%–70% probability of functional speech decline and over 90% probability of subclinical articulatory decline (e.g., changes in jaw and lip motor performance) (101). Thus, we consider the chance of the participants in the ALS − B subgroup having subclinical bulbar involvement to be high. The observed differences in the composite outcome measures between ALS − B and healthy controls corroborate this presumption, demonstrating that (1) the majority if not all of the participants in the ALS − B subgroup had already experienced subclinical bulbar involvement and (2) our multimodal measurement tool can effectively capture such subclinical changes. Second, our participants spanned a wide age range, and age could influence some speech features such as vocal pitch. This potential confounder should be further investigated and factored into the analysis in future research. Third, the dataset for validating the proposed multimodal measurement tool consisted of multiple samples collected from each participant. As an initial effort of validation, we did not differentiate between- and within-participant variance during the training of the ML models. While all models were tested with 5-fold cross-validation repeated 10 times to reduce the possibility of overfitting to data samples drawn from specific participants, future larger-scale validation studies should account for the potential between- and within-participant effects (e.g., by testing different data partitioning methods such as leave-one-subject-out).

## 5 Conclusion

Using combined facial sEMG and acoustic instrumental techniques coupled with a fit-for-purpose data analytic algorithm, an objective multimodal measurement tool was developed to link seven theoretical constructs of bulbar/speech motor control (prosody, pause, functional connectivity, amplitude, rhythm, complexity, regularity) with measurable outcomes. Based on validation testing on over 400 speech samples collected from a heterogeneous group of individuals with ALS and age-matched healthy controls, the multimodal measurement tool demonstrated (1) internal consistency and structural validity for measuring the targeted constructs and (2) concurrent validity in relation to established clinical and functional criteria for diagnosing and evaluating bulbar involvement in ALS. Moreover, all outcomes of the measurement tool exhibit interpretable disease-related changes in line with the neuromuscular pathology of bulbar involvement in ALS. Together, these outcomes show high efficacy for detecting subclinical bulbar changes, both during the prodromal stage and during the transition from prodromal to symptomatic stages, and outperform the unimodal subcomponents as obtained from each constituent modality. The findings of this study provide compelling initial evidence for the utility of the multimodal measurement tool for improving both the early diagnosis and progress monitoring of bulbar involvement, which are critical for timely access to and delivery of optimal clinical care. From a practical perspective, the multimodal measurement tool has several desirable features, including the use of noninvasive, clinically readily available instruments, fully automated data analytics, and mechanistically relevant and clinically interpretable outcomes, enhancing its scalability into future clinical settings.

## Data availability statement

The datasets presented in this article are not readily available because the raw data include audio recordings of speech, which contain potentially identifiable information about the participants. Requests to access the datasets should be directed to PR, prong@ku.edu.

## Ethics statement

The studies involving humans were approved by University of Kansas Medical Center Institutional Review Board. The studies were conducted in accordance with the local legislation and institutional requirements. The participants provided their written informed consent to participate in this study.

## Author contributions

PR: Conceptualization, Data curation, Formal analysis, Funding acquisition, Investigation, Methodology, Project administration, Resources, Software, Supervision, Validation, Visualization, Writing – original draft, Writing – review & editing. LH: Methodology, Resources, Writing – review & editing. GP: Methodology, Writing – review & editing.
