# Supplementary material for: A multimodal approach to automated hierarchical assessment of bulbar involvement in amyotrophic lateral sclerosis
Source: Front Neurol. 2024 May 21;15:1396002. doi: 10.3389/fneur.2024.1396002 (PMC11148322; doi:10.3389/fneur.2024.1396002)
Supplement: Supplementary file 1 [file Table_1.DOCX]

Table S1. Demographic, clinical, and functional speech characteristics of participants. Descriptive statistics are provided as mean (standard deviation) by group at the bottom of the table. Gender: M = male, F = female. Onset: B = bulbar, C = cervical, L = lumbar, N = neck. DaysSinceDiag = disease duration in days since diagnosis. SIT_Intell = speech intelligibility in percentage of intelligible words, as assessed by the Sentence Intelligibility Test (SIT). SIT_SR = speaking rate in words per minute (WPM), as assessed by the SIT. Group: Control = healthy controls; ALS-B = individuals at the prodromal stage of bulbar involvement secondary to amyotrophic lateral sclerosis (ALS), absent of overt clinical bulbar symptoms; ALS+B = individuals at the symptomatic stage of bulbar involvement secondary to ALS, presented with overt clinical bulbar symptoms. All participants were nonsmokers, who either had no smoking history or quit smoking for more than one year.

| **SubjectID** | **Gender** | **Age** | **Onset** | **DaysSinceDiag** | **SIT_Intell** | **SIT_SR** | **Group** |
| --- | --- | --- | --- | --- | --- | --- | --- |
| ALS1 | M | 73 | B | 134 | 54.55 | 102.99 | ALS+B |
| ALS2 | M | 47 | C | 1668 | 99.55 | 150.35 | ALS-B |
| ALS3 | M | 74 | C/N | 87 | 92.73 | 128.27 | ALS-B |
| ALS4 | M | 72 | L | 407 | 95.91 | 122.79 | ALS-B |
| ALS5 | F | 65 | B | 266 | 95.00 | 85.14 | ALS+B |
| ALS6 | M | 58 | L | 56 | 96.82 | 190.78 | ALS-B |
| ALS7 | F | 66 | C | 294 | 97.27 | 146.62 | ALS+B |
| ALS8 | F | 62 | B | 150 | 14.09 | 47.64 | ALS+B |
| ALS9 | M | 38 | L | 123 | 97.27 | 152.78 | ALS+B |
| ALS10 | M | 38 | C/L | 491 | 99.55 | 154.24 | ALS-B |
| ALS11 | M | 52 | C | 192 | 99.09 | 149.45 | ALS-B |
| ALS12 | F | 73 | B | 571 | 94.55 | 121.77 | ALS+B |
| ALS13 | F | 56 | C | 269 | 99.09 | 202.45 | ALS-B |
| HC1 | F | 55 |  |  | 100 | 188.19 | Control |
| HC2 | F | 38 |  |  | 100 | 231.78 | Control |
| HC3 | M | 65 |  |  | 99.55 | 167.10 | Control |
| HC4 | M | 76 |  |  | 99.55 | 196.46 | Control |
| HC5 | M | 81 |  |  | 100 | 166.04 | Control |
| HC6 | F | 71 |  |  | 100 | 186.45 | Control |
| HC7 | F | 80 |  |  | 98.64 | 192.15 | Control |
| HC8 | F | 62 |  |  | 99.09 | 171.36 | Control |
| HC9 | F | 74 |  |  | 98.64 | 146.21 | Control |
| HC10 | F | 66 |  |  | 99.09 | 190.75 | Control |
| Control |  | 66.80 (13.02) |  | - | 99.45 (0.56) | 183.65 (23.02) |  |
| ALS-B |  | 56.71 (12.92) |  | 452.86 (558.77) | 97.53 (2.56) | 156.90 (29.75) |  |
| ALS+B |  | 62.83 (12.95) |  | 256.33 (170.05) | 75.45 (34.35) | 109.49 (39.66) |  |
